# Replication-Competent Infectious Hepatitis B Virus Vectors Carrying Substantially Sized Transgenes by Redesigned Viral Polymerase Translation

**DOI:** 10.1371/journal.pone.0060306

**Published:** 2013-04-02

**Authors:** Zihua Wang, Li Wu, Xin Cheng, Shizhu Liu, Baosheng Li, Haijun Li, Fubiao Kang, Junping Wang, Huan Xia, Caiyan Ping, Michael Nassal, Dianxing Sun

**Affiliations:** 1 The Liver Disease Diagnosis and Treatment Center of PLA, Bethune International Peace Hospital, Shijiazhuang, PR China; 2 The Third Military Medical University, Chongqing, PR China; 3 University Hospital Freiburg, Internal Medicine II/Molecular Biology, Freiburg, Germany; Yonsei University, Republic of Korea

## Abstract

Viral vectors are engineered virus variants able to deliver nonviral genetic information into cells, usually by the same routes as the parental viruses. For several virus families, replication-competent vectors carrying reporter genes have become invaluable tools for easy and quantitative monitoring of replication and infection, and thus also for identifying antivirals and virus susceptible cells. For hepatitis B virus (HBV), a small enveloped DNA virus causing B-type hepatitis, such vectors are not available because insertions into its tiny 3.2 kb genome almost inevitably affect essential replication elements. HBV replicates by reverse transcription of the pregenomic (pg) RNA which is also required as bicistronic mRNA for the capsid (core) protein and the reverse transcriptase (Pol); their open reading frames (ORFs) overlap by some 150 basepairs. Translation of the downstream Pol ORF does not involve a conventional internal ribosome entry site (IRES). We reasoned that duplicating the overlap region and providing artificial IRES control for translation of both Pol and an in-between inserted transgene might yield a functional tricistronic pgRNA, without interfering with envelope protein expression. As IRESs we used a 22 nucleotide element termed Rbm3 IRES to minimize genome size increase. Model plasmids confirmed its activity even in tricistronic arrangements. Analogous plasmids for complete HBV genomes carrying 399 bp and 720 bp transgenes for blasticidin resistance (BsdR) and humanized *Renilla* green fluorescent protein (hrGFP) produced core and envelope proteins like wild-type HBV; while the hrGFP vector replicated poorly, the BsdR vector generated around 40% as much replicative DNA as wild-type HBV. Both vectors, however, formed enveloped virions which were infectious for HBV-susceptible HepaRG cells. Because numerous reporter and effector genes with sizes of around 500 bp or less are available, the new HBV vectors should become highly useful tools to better understand, and combat, this important pathogen.

## Introduction

Chronic infection with hepatitis B virus (HBV) affects up to 400 million people worldwide, putting them at an increased risk to develop liver fibrosis, cirrhosis and hepatocellular carcinoma [1]. Current therapies, using type-I interferon or nucleos(t)ide analogs, are only partially effective [2]. Finding new treatment strategies is hampered by experimental limitations [3]; due to HBV's liver tropism and narrow host range, restricted to humans and the Great Apes, primary hepatocytes from humans and (for poorly understood reasons) from tupaias [4] have long remained the only cell culture infection system; more recently, a single human hepatoma cell line, HepaRG, has shown to be susceptible to HBV infection upon differentiation [5]. Hence the early steps of infection are still poorly understood, including the identity of the cellular receptors. Viral replication, in contrast, is known in considerable detail from genetic studies in transfected cells and from biochemical reconstitution of some key replication steps (for reviews: [6], [7]). As outlined below, overall these data revealed an intricate interplay between the few viral gene products and numerous cis-elements, streamlined to warrant function of the tiny (3.2 kb) and extremely compactly organized HBV genome which therefore is exquisitely sensitive to sequence manipulations.

For various other virus families, including important pathogens like human immunodeficiency virus 1 (HIV-1) and hepatitis C virus (HCV), it has been possible to engineer artificial variants carrying non-viral information, e.g. genes for reporter or marker proteins, without compromising replication competence [8], [9], [10]. Usually, such viral vectors exploit the same routes into target cells and show the same host dependence for replication as their parental viruses. Infection- and/or replication-dependent expression of the vector-encoded reporter transgene thus greatly facilitates monitoring the route of infection as well as infection and replication efficiency and their dependence on host factors [11], [12]. Furthermore, the much simplified quantitative assessments enable efficient screening for inhibitors [13] and also the identification of virus-susceptible cells.

Due to the peculiarities of HBV´s genome organization and replication strategy, development of replication-competent HBV-based vectors has met with serious difficulties. In HB virions, the genome is present mostly as a relaxed-circular (RC) molecule (and to a lesser extent as a double-stranded linear (dsL) DNA) in which one of the DNA strands is covalently linked to the viral polymerase [6]. Upon infection, the RC-DNA is converted into covalently closed circular (ccc) DNA which serves as transcription template. The genome contains four widely overlapping open reading frames (ORFs), namely preS1/preS2/S (encoding the three C terminally collinear envelope or surface proteins L, M and S), preC/C (encoding the capsid or core protein, and the nonessential precore protein giving rise to the secretory hepatitis B e antigen [HBeAg]), X (encoding HBx, a transcriptional activator required for establishment of infection [14], [15], [16]), and P (encoding the viral polymerase (Pol), a multidomain enzyme with reverse transcriptase, RNase H and protein-priming activities; [7]). The P ORF encompasses nearly 80% of the viral genome and overlaps with all other ORFs. Expression of the gene products is achieved via four classes of transcripts from internal promoters. Particularly important is the pregenomic (pg) RNA, a greater-than-unit length transcript which acts as bicistronic mRNA for the core protein and Pol [17], and in addition as template for reverse transcription into progeny DNA genomes. By necessity, the promoter and enhancer sequences overlap with coding information and so do the numerous cis-elements, including but not limited to the encapsidation signal ε and the direct repeat sequences DR1 and DR2, which are indispensable for the packaging of pgRNA into new capsids and its reverse transcription into RC-DNA. Almost inevitably, interrupting this intricate arrangement at any point by insertion of foreign sequence is detrimental to viral replication.

Consequently, most previous efforts have been restricted to replication-defective HBV vectors in which the destroyed functions must be provided in *trans*, i.e. by a cotransfected helper plasmid. Such vectors are capable of just a single infection round; this may be a desired safety feature in gene therapy but it prevents replication-related applications. A second restriction is posed by the defined inner volume of the icosahedral capsid which limits the length of packagable RNA and DNA. Rather than adding extra information, the commonly used approach was therefore to replace authentic viral sequences, e.g. the S ORF [18], [19], [20], [21] or the C ORF [22] with the transgene of interest. Some of these studies have indeed documented liver-specific gene transfer by such vectors; our own recent work, employing a chimeric replication-defective adenovirus-HBV vector carrying a truncated matrix-metalloproteinase-8 (MMP-8) has shown substantial therapeutic benefit in a rat model of liver cirrhosis [23].

Only two published studies report attempts to generate replication-competent HBV vectors. In one study, a 276 bp sequence encoding the HIV-1 Tat protein was inserted in-frame into the spacer region connecting the Terminal Protein (TP) domain of Pol to its reverse transcriptase (RT) domain [24]; the spacer is dispensable for Pol function but overlappingly encodes the PreS1 region of the L protein which is essential for formation of infectious virions. Upon transfection, Tat-dependent transactivation was observed yet formation of replicative HBV DNA was drastically (>95%) reduced. Infectivity was not experimentally addressed, and probably lost due to lack of PreS1. The second study introduced 63 bp of foreign DNA into an HBV construct lacking the X gene, thereby also destroying the direct repeat DR2. Reportedly, a low level of viral DNA was observed in Huh7 cells but not in HepG2 cells, two human hepatoma cell lines which both support wild-type HBV replication (but are not infectable). Because DR2 is critical for RC-DNA formation and HBx is required for infection [15], [16], proper replication and infectivity of such constructs is highly questionable.

Hence we considered an alternative vector design, the key feature of which is to uncouple core protein and Pol expression to convert the authentic bicistronic pgRNA into a tricistronic derivative capable of directing translation of a third, heterologous gene while remaining competent for being encapsidated and reverse transcribed. This may also leave the preS1 and preS2/S promoters within the P ORF intact, giving rise to the entire set of envelope proteins and thus allowing formation of infectious recombinant virions. On authentic pgRNA the core and Pol ORFs overlap by some 150 nucleotides (nt). Importantly, this region does not contain (on the RNA and DNA level) any known cis-elements except those controlling translation initiation of the downstream Pol ORF. The Pol initiation codon resides, in a different frame, in the 3′ part of the core ORF proteins. Translation initiation likely involves termination–reinitiation at an upstream minicistron [25] rather than a conventional internal ribosome entry site (IRES) as present in some other viruses (for review, see [26]).

One obvious prerequisite for uncoupling translation of the two viral proteins was to duplicate the overlap region to create two independent, complete ORFs. A second was to devise an artifical translation control mechanism for Pol expression. Notably, authentic translation results in lower levels of Pol than of core protein because only one Pol molecule is required for being copackaged with one pgRNA molecule per 240 subunit capsid [27]; hence also the artificial substitute would not have to be exceedingly efficient. A third premise was that the new control elements require little genetic space. In vitro assembly of RNA-filled capsids, in the absence of Pol, has suggested about two equivalents of pgRNA (∼7 kb) as an upper limit [28], [29]. Conversion into double-stranded DNA would substantially increase the space requirements [30], [31]. Lastly, the envisaged tricistronic pgRNA arrangement with a transgene inserted between the separated core and Pol ORFs must accomodate additional translational control elements for the transgene.

One potential realization would be fusions with "self-cleaving" 2A peptides which probably act by ribosome-internal termination-reinitiation [32]; however, while only about 20 amino acid (aa) long, the 2A peptides remain on the upstream encoded protein and thus might interfere with proper function. A second option are IRESs which direct 5′ cap-independent translation initiation. A prominent example is the IRES from encephalomyocarditis virus (EMCV) [33], [34], [35]. However, with about 450 nt in length a single EMCV IRES would already occupy a substantial fraction of the limited space on the recombinant vector genome; the same holds for most of the other well-established viral IRES elements [26]. Therefore, the reported IRES activity of some very short sequences, including a 22 nt sequence originally assigned to the RNA-binding motif protein 3 (Rbm3) mRNA [36], appeared as a most promising alternative. Although its being a genuine part of the Rbm3 mRNA may require further investigation ([37]; and V. Mauro, personal communication) it has successfully been used to express downstream cistrons in different cell lines from different vectors [36], [37], [38]. The mechanism of ribosome recruitment by the Rbm3 IRES (for sake of simplicity we will use this term throughout) and some other small elements is debated; for instance, the element was proposed to act by introducing an artificial 3′ splice acceptor, thus giving rise to monocistronic mRNAs for the downstream cistron [37]. However, this was not confirmed in a recent study [39]. Furthermore, we reasoned that substantial formation of such aberrant splice products would be detectable by Northern blot analyses, and be accompanied by a corresponding lack of full-length recombinant pgRNA and its potential reverse transcription products.

The data reported below demonstrate that HBV genomes redesigned to specify tricistronic pgRNAs, with a transgene inserted between separated core and Pol ORFs and transgene and Pol expression controlled using two Rbm3 IRES elements, are indeed able to replicate and form infectious virions. Although replication efficiency dropped with increasing transgene size, a vector carrying a 399 bp transgene replicated at about 40% the level of wild-type HBV, and the low level of replication induced by a 720 bp transgene was still sufficient to easily detect infected cells via expression of the encoded fluorescent reporter protein and even infection inhibition by neutralizing antibodies. Equipped with appropriate transgenes, the reported replication-competent HBV vectors should become highly useful novel tools to unravel aspects of the HBV infectious cycle that thus far remained refractory to analysis.

## Results

### The 22-nt Rbm3 IRES element directs expression of the downstream cistron in bicistronic and tricistronic HBV pgRNA-like model RNAs

As yet there are no reports on the translation initiation efficiency of the Rbm3 IRES in human Huh7 and HepG2 hepatoma cells. To explore its principal suitability for expressing a downstream cistron from bicistronic and, as eventually intended, tricistronic HBV pgRNA-like transcripts two series of model plasmids were constructed which mimic typical features of the HBV pgRNA. For the bicistronic constructs, analogous plasmids containing instead the EMCV IRES served as reference. Lastly, we included constructs in which the reporter gene was directly fused to the authentic Pol start to test for background (i.e. Rbm3 IRES-independent) translation of the transgene via potentially present endogenous translation control signals in the preceding core ORF region.

**Figure 1:**
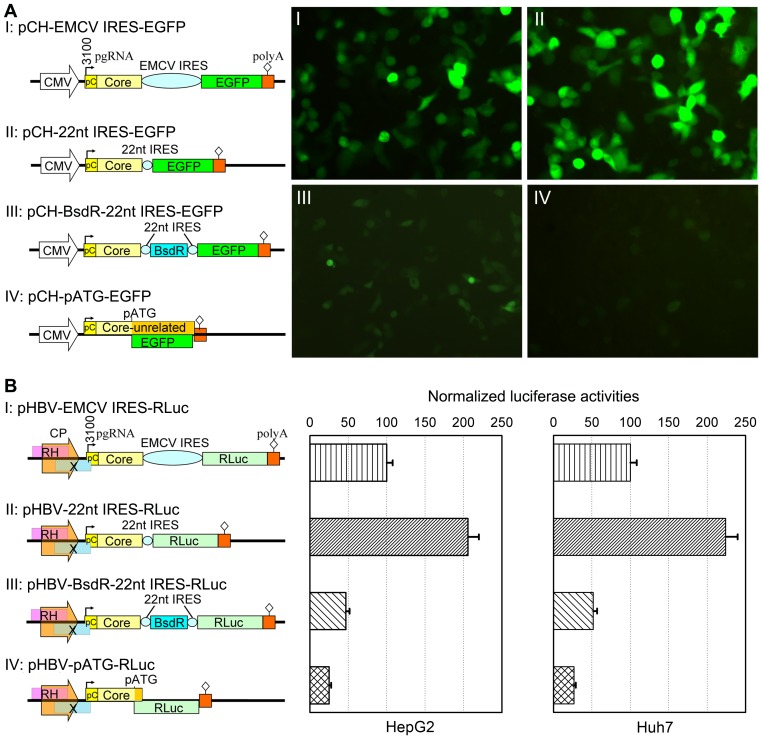
The 22-nt Rbm3 IRES directs downstream reporter gene expression from bicistronic and tricistronic HBV pgRNA mimics. (A) EGFP as reporter. *Left*: Schematic representation of the expression cassettes used. The upstream part is identical to the HBV vector pCH-9/3093 [40], [21] in which the CMV-IE promoter drives transcription of authentic pgRNA starting at nt position 3100. In constructs I - III the sequence following the end of the core gene was replaced by the indicated control elements and a gene for EGFP; in construct IV, the EGFP gene was fused to the authentic Pol translation start. pC denotes the preC region; its start codon (nt positions 3096–3098) is not part of the pgRNA. BsdR, blasticidin resistance. *Right*: EGFP fluorescence in HepG2 cells transfected with constructs I - IV on day 4 post transfection. All micrographs were taken under identical conditions. Comparable results were obtained in Huh7 cells. (B) RLuc as reporter. The constructs are based on the HBV expression vector pHBV1.3 [46] in which pgRNA transcription is driven by the authentic HBV core promoter which overlaps with coding information for the RNase H (RH) domain of Pol and HBx (X), and which also generates precore mRNA, thus giving rise to genuine HBeAg. No significant differences in HBeAg levels between constructs I, II, and III were detected by HBeAg ELISA (n = 3; *P*>0.05). Histograms on the right show relative RLuc activities in HepG2 and Huh7 cells compared to firefly luciferase activities from cotransfected pGL3 control vector, as determined by the dual luciferase assay. Values for the EMCV IRES construct were set as 100%. Mean values from three independent experiments are shown; error bars indicate standard deviation (SD). All differences between the groups were significant, with *P*<0.01 for all pairs except III vs. IV (*P*<0.05).

The first panel of plasmids (Fig. 1A), driven by the strong CMV-IE enhancer/promoter, contained the same 5′ HBV sequence as the HBV expression vector pCH-9/3093 [40], [41] except that the sequence downstream of the 3′ end of the core gene was replaced by the respective IRES element plus a gene for enhanced green fluorescent protein (EGFP; Fig. 1A, constructs I and II); this allowed estimating relative IRES activity by visual inspection of the GFP fluorescence; for the tricistronic arrangement (construct III), a 399 bp blasticidin resistance (BsdR) gene with its own 5′ Rbm IRES was inserted between the core and EGFP genes. Construct IV contained the EGFP gene directly fused to the authentic Pol initiation codon, causing the genuine core protein sequence to encompass only the first 135 aa. To account for potential differences in transfection efficiency, the culture supernatants were quantitatively monitored for core-derived products with HBe cross-reactivity by HBeAg ELISA as a surrogate marker for core protein expression [42], [40]. However, compared to the values obtained for construct I (set as 100%), no significant differences to those for constructs II and III were seen in both HepG2 and Huh7 cells. For HepG2 cells, the values for constructs I, II and III were 100.00±6.34%, 96.05±9.33%, and 96.71±5.92% (n = 3; *P*>0.05), and for Huh7 cells 100.00±6.34%, II:96.05±9.33%, and 96.71±5.92% (n = 3; *P*>0.05), respectively. For the bicistronic constructs, EGFP fluorescence from the Rbm3 IRES was stronger in both hepatoma lines than that from the corresponding EMCV IRES construct, as shown for HepG2 cell in Fig. 1A (panel II vs. I). EGFP fluorescence from the tricistronic construct (Fig. 1A, panel III) was substantially reduced yet remained easily detectable, clearly exceeding the signals from construct IV (Fig 1A, IV).

Because CMV is a very strong promoter, we next tested whether the endogenous HBV core promoter/enhancer II (which results in about 3-fold less pgRNA compared to the CMV promoter [43]) would lead to detectable expression of the downstream cistron. To allow for a quantitative assessment, the EGFP gene was replaced by the Rluc gene (Fig. 1B). The RLuc constructs were cotransfected with the firefly luciferase vector pGL3-Control (Promega) and relative RLuc vs. firefly luciferase activity was monitored using the dual luciferase luminometric assay (Promega). Both HepG2 and Huh7 cells gave very similar results. Relative RLuc activity from the Rbm3 IRES was more than 2-fold higher than from the EMCV IRES (Fig 1B, II vs. I). RLuc activity from the tricistronic construct was reduced (Fig. 1B, III) but still about half as high as from the bicistronic EMCV IRES construct, and it clearly exceeded that from the construct having the RLuc gene fused to the authentic Pol start (Fig 1B, IV). In HepG2 cells the numerical values were for construct I, 100.00±7.73%; for II, 205.90±14.00%; for III, 46.82±4.86%; and for IV, 25.07±2.53%. In Huh7 cells the values were for construct I, 100.00±8.24%; for II, 223.88±15.74%; for III, 51.69±4.88%; and for IV, 26.52±2.35%. All differences between the groups were significant, with *P*<0.01 for all pairs except III vs. IV (*P*<0.05).

Consistent with the EGFP fluorescence data these results indicated that, even if driven by the authentic HBV core promoter, the Rbm3 IRES directed higher levels of reporter gene expression in human hepatoma cells than the EMCV IRES from bicistronic constructs; importantly, substantial expression of the most downstream cistron was also achieved from the tricistronic constructs mimicking pgRNA with a transgene inserted between core and Pol ORF.

### Construction of replication-competent HBV vectors

**Figure 2:**
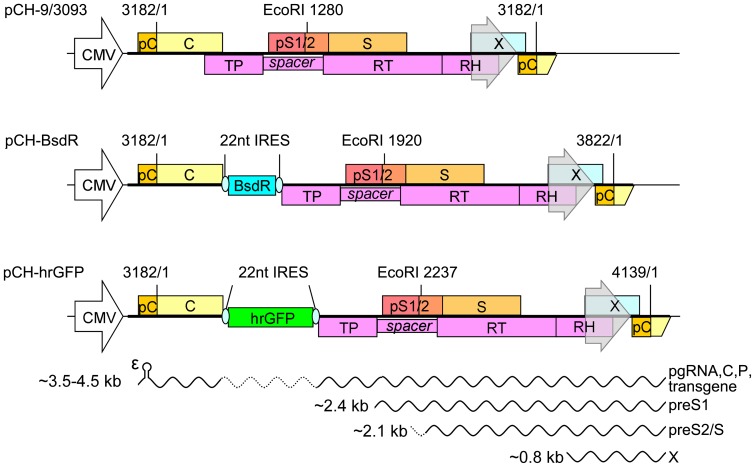
Genomic organization and transcripts of HBV and recombinant HBV vectors. The parental plasmid pCH-9/3093 is based on a 1.05× HBV genome (thick black line, HBV nucleotides 3093 to 3182-1 plus 3182/1 to 84) in which the CMV promoter replaces the endogenous HBV core promoter (transparent grey arrowhead close to the 3′ end) that drives pgRNA transcription on the authentic, circular DNA genome. Numbers are nt coordinates relative to the core ORF start [75]; the Eco RI site at position 1280 is the reference point in an alternative numbering system [76]. ORFs are indicated by boxes: pC, precore; C, core; pS1/2 and S, preS1, preS2, and S domains of the envelope proteins, respectively; X, HBx; TP, RT, and RH, terminal protein, reverse transcriptase, and RNase H domains of Pol (P); hrGFP, humanized *Renilla* GFP. RNAs (bottom) are indicated as sinusoidal lines. Authentic pgRNA is about 3.5 kb in length, encodes core protein and Pol, and carries the 5′ proximal encapsidation signal ε which mediates, via Pol binding, specific encapsidation of the pgRNA and initiation of reverse transcription. The subgenomic 2.4 kb (preS1) and 2.1 kb (preS2/S) transcripts for the L, and the M and S envelope proteins and the 0.8 kb HBx RNA are generated from internal promoters located in the Pol ORF. The recombinant genomes are expected to generate size-increased pgRNAs (symbolized by the inserted dashed sinusoidal line) plus unmodified subgenomic RNAs.

Based on the above described results we chose the tricistronic arrangement as present in constructs III, i.e. with two Rbm3 IRES elements, for generating potentially replication competent transgene-carrying HBV vectors. Accordingly, in the HBV expression vector pCH-9/3093 (Fig. 2, top) the about 150 bp sequence overlappingly encoding the C terminal part of the core protein and the N terminal part of Pol was duplicated, and in-between the sequences for BsdR (399 bp) or hrGFP (720 bp), flanked by an upstream copy (for transgene expression) and a downstream copy of the Rbm3 IRES (for Pol expression) were inserted (Fig. 2). In addition, the codon for the second Pol amino acid was mutated from Pro (CCC) to Ala (GCC) to conform to the Kozak consensus sequence which asks for a G at position +4 relative to the start ATG for optimal translation initiation [44]. The exact junction sequences upstream and downstream of the IRES elements are given in Table S2. Together, the heterologous sequences increase the size of the unit length HBV genome from 3182 bp to 3822 bp in pCH-BsdR, and to 4139 bp in pCH-hrGFP. These size differences should allow for an estimate as to how much heterologous sequence the modified HBV genome tolerates without losing replication competence. A schematic view of the major transcripts expected to be produced from these constructs is shown at the bottom of Fig. 2.

### Evaluation of gene expression from the recombinant HBV vector constructs

**Figure 3:**
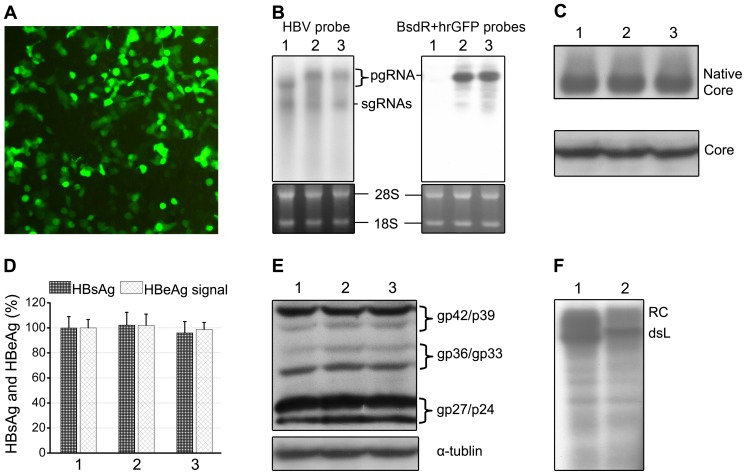
Evaluation of gene expression from the recombinant HBV vectors. HepG2 cells were transfected with the wild-type HBV plasmid pCH-9/3093 (1), or the recombinant vector plasmids pCH-BsdR (2) or pCH-hrGFP (3). (A) Expression of the hrGFP transgene detected by immuno fluorescence. (B) HBV related transcripts. Total cytoplasmic RNA from the transfected cells was analyzed by Northern blotting using α ^32^P-labeled HBV probe (left panel) or a 1∶1 mixture of BsdR- and hrGFP-specific probes (right panel). The positions of the pgRNA and subgenomic (sg) RNAs are indicated. Ethidium bromide staining of 28 S and 18 S rRNA confirmed equal loading and intactness of the RNAs. No products attributable to major aberrantly spliced RNAs were detected by either probe. (C) Wild-type HBV-like production of core protein and capsids. Equal aliquots from cytoplasmic lysates were analyzed by immunoblotting with core-specific antibodies after NAGE (top; intact capsids) or after SDS-PAGE (bottom; core protein) and chemiluminescent detection. (D) HBsAg and HBeAg ELISA. Levels of secreted HBsAg and of HBeAg as surrogate marker for core protein were determined by ELISA and normalized those from the wild-type HBV plasmid pCH-9/3093 which were set as 100%. Values are the mean of three independent transfections; error bars indicate SD. Differences between groups were not significant (*P*>0.05). (E) Wild-type HBV-like production of L, M and S proteins. Immunoblotting after SDS-PAGE was performed using antibody 4/7B which recognizes a linear epitope in the S protein which thus is also contained in M and L proteins [71]. The positions of glycosylated and non-glycosylated L (gp42/p39) and S protein (gp27/p24), and of di-glycosylated and mono-glycosylated M protein (gp36/gp33) are indicated. Immunodetection of tubulin served as a loading control. (F) pCH-BsdR generates functional Pol. Cytoplasmic core particles from cells transfected with pCH-9/3093 and pCH-BsdR were subjected to endogenous polymerase reaction conditions including [α-^32^P]dCTP. Purified DNAs were separated by agarose gel electrophoresis, and radiolabeled products were detected by autoradiography. The positions of RC- and dsL DNA are indicated.

As a preliminary test for transgene expression from the new constructs we transfected the pCH-hrGFP vector into HepG2 cells and assessed hrGFP expression by fluorescence microscopy, which confirmed high level hrGFP expression (Fig. 3A). Expression of functional BsdR was subsequently demonstrated by formation of stable Bsd resistant cell clones upon transfection of pCH-BsdR into HepG2 cells (Ping, Cheng, Sun, unpublished data). To examine the RNAs giving rise to the transgene products we transfected both vectors in parallel with the parental wild-type HBV expression plasmid into HepG2 cells and performed Northern blotting. An HBV-specific probe (Fig. 3B, left panel) revealed the presence of recombinant pgRNAs with an increased size compared to wild-type pgRNA plus subgenomic RNAs with the same mobility as those produced by wild-type HBV, both as expected. The levels of all RNAs were similar. To directly demonstrate the presence of the transgenes in the recombinant pgRNAs, a parallel blot was analyzed using a 1∶1 mixture of hrGFP- and BsdR-specific probes (Fig. 3B, right panel). Signals were only detected in the samples from the transgene vector-transfected cells (lanes 2 and 3), and the dominant bands appeared at the same position as the recombinant pgRNAs detected by the HBV probe. Notably, no major extra products indicative of aberrantly spliced transgene transcripts were seen. Hence transgene expression must largely have occurred from the recombinant pgRNAs, making it unlikely that the Rbm3 IRES element functions by promoting splicing.

Next we investigated formation of the viral structural proteins. In all these experiments samples from mock transfected cells gave no signals above background (data not shown). Production of similar levels of core protein and assembled capsids from all three vectors was shown by immunoblotting after SDS-PAGE (Fig. 3C bottom) and after NAGE (Fig. 3C top), respectively. Comparable core protein production was further confirmed by surrogate HBeAg ELISA (Fig. 3D, bars labeled HBeAg signal). HBsAg ELISAs (Fig. 3D, bars labeled HBsAg) revealed similar levels of surface antigen in the culture supernatants from cells transfected with all three vectors. None of the small differences between the groups were significant (n = 3; *P*>0.05). However, the HBsAg ELISA does not discriminate between the three envelope proteins L, M and S; because especially the Sp1 promoter for the L protein mRNA, located between nt positions 810–908 (corresponding to positions 2710–2808 in the Eco RI site based numbering system [45]), is not far from the transgene insertion site and L protein is essential for infectivity, we used immunoblotting to directly test for the presence and amounts of the three proteins. Indeed, similar levels of L, M and S in their differentially glycosylated forms (gp42/p39, gp36/gp33 and gp27/p24) as from wild-type HBV were produced from the recombinant vectors (Fig. 3E). As direct Pol detection by Western blotting is difficult and does not discriminate between functional and non-functional Pol molecules, we next used the endogenous polymerase reaction (EPR) to detect its presence. In the EPR, Pol present in nucleocapsids extends, upon provision of dNTPs, DNA strands resulting from reverse transcription of co-packaged pgRNA. To this end, intracellular core particles from wild-type HBV or BsdR vector transfected HepG2 cells were incubated with dNTPs including [α-^32^P]dCTP, and radiolabeled DNA products were separated by gel electrophoresis and visualized via autoradiography (Fig. 3F). Products with mobilities expected for RC and dsL DNA were easily detectable from the recombinant vector, albeit at somewhat reduced levels. Importantly, the recombinant replicative DNAs exerted a reduced mobility, as expected from the inserted transgene. These data demonstrated that functional Pol was translated from the BsdR vector and that the size-increased pgRNA was packaged and reverse transcribed. The hrGFP vector generated only very weak signals (not shown) yet a low level of replication was subsequently confirmed by Southern blotting (see below).

### Assessment of HBV vector replication by Southern blotting and qPCR

**Figure 4:**
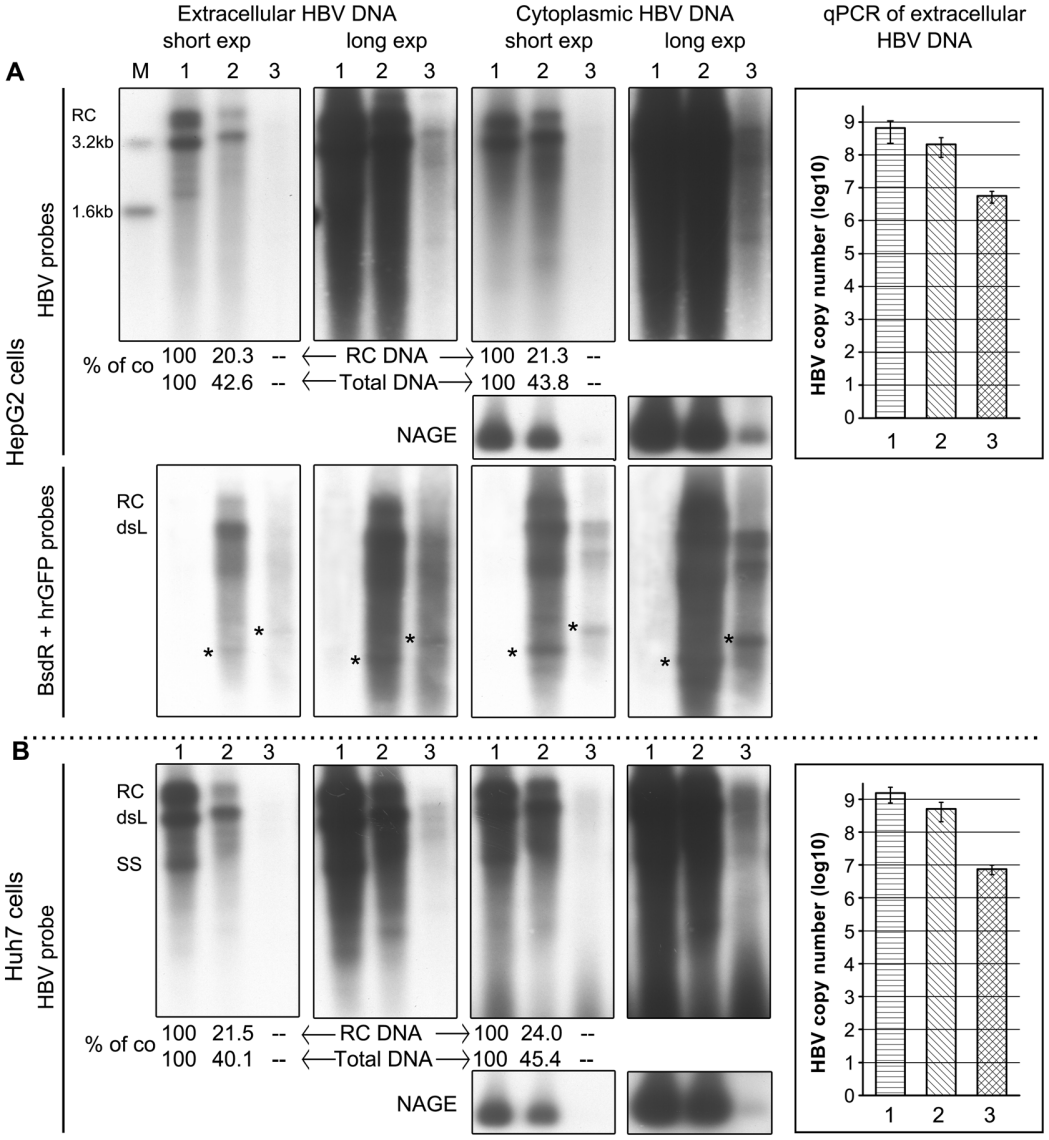
Replication efficiency of BsdR and hrGFP HBV vectors. HepG2 cells (A) or Huh7 cells (B) were transfected with plasmid pCH-9/3093 (1), pCH-BsdR (2) or pCH-hrGFP (3). Extracellular and cytoplasmic HBV DNA was extracted 4 days post-transfection. Replicative intermediates were monitored by Southern blotting using α ^32^P-labeled HBV probe, or a mixture of BsdR- and hrGFP-specific probes (A, lower panel). The positions of RC- and dsL HBV DNA are indicated. To reveal the weak signals from the pCH-hrGFP construct, long exposures of the same blots are also shown. For the BsdR construct, the signals for total DNA, and separately for RC-DNA were quantitated by phosphorimaging and related to the signal intensities from the wild-type HBV samples (set at 100%); mean values from three experiments are indicated at the bottom of the blots. Both the total amount of replicative DNA and RC-DNA signal intensity were significantly different between wild-type constructs and BsdR in both HepG2 and Huh7 cells (*P*<0.01). The transgene-specific probes (A, lower panels) detected additional faster migrating species in the transgene-related samples, including distinct bands whose mobility slightly differed between BsdR and hrGFP vector (labeled with asterisks); these might represent reverse transcription products from small amounts of spliced recombinant pgRNAs (see text for details). In addition, bulk viral DNA associated with capsids was visualized by hybridization with a ^32^P-labeled HBV DNA probe following separation of the capsids by NAGE (small panels). The qPCR results for extracellular HBV DNA are shown in the bar graphs on the right. Copy numbers are given as log10 per 1 ml of culture supernatant, collected from 72 h–96 h post transfection. The mean values from three independent experiments including SD are shown. All differences were significant, with *P*<0.05 for BsdR vector vs. wild-type HBV vector, and *P*<0.01 for the others.

Next, formation of replicative DNA intermediates in intracellular and extracellular viral particles was directly analyzed by Southern blotting, and the amounts of viral genomes in the culture supernatants were determined by quantitative PCR (qPCR). As shown in Fig. 4, the HBV-specific probe produced a similar band pattern for the BsdR vector, both in HepG2 and Huh7 cells, as the RC-DNA and dsL-DNA signals from the wild-type HBV construct, except that both migrated more slowly, as expected from the presence of the additional transgene. The total amount of replicative DNA was reduced to about 40–45% compared to wild-type (n = 3; P<0.01 by Student's t-test), as corroborated by the relative signal intensities obtained for encapsidated DNA following NAGE analysis of intracellular capsids (panels labeled NAGE). RC-DNA signal intensity was more pronouncedly reduced to about 20–25% (*P*<0.01 by Student's t test) of the wild-type level.

Although cells transfected with the pCH-hrGFP construct showed strong green fluorescence (Fig. 3A), only small amounts of replicative DNA intermediates were detectable upon long exposure of the Southern blots (Fig. 4A, B, panels labeled "long exp"). This was also reflected in the very weak signals obtained for encapsidated DNA following NAGE analysis (Fig. 4A, B, small panels labeled "NAGE"). The largest distinct Southern blot band migrated slightly slower than the dsL product from the BsdR vector, consistent with its representing dsL-DNA rather than RC-DNA of the hrGFP vector. To directly demonstrate the presence of transgene sequences in the recombinant viral DNAs, parallel blots were analyzed using a mixture of BsdR- and hrGFP-specific probes (Fig. 4A, lower panel). As expected, no signals occurred with the wild-type HBV vector transfected samples (lanes 1), confirming that the signals from the transgene vector samples (lanes 2 and 3) were specific. Their relative intensities were similar to those detected with the HBV-specific probe; however, products migrating faster than dsL-DNA appeared somewhat more pronounced, including a distinct fast species that slightly differed in size for the BsdR versus hrGFP vector (labeled with asterisks). As these bands were not detected by the HBV probe, they might represent transgene-comprising reverse transcription products derived from small amounts of spliced RNAs. Even though such RNAs were not evident by Northern blotting, for wild-type HBV shorter, splicing-derived pgRNAs are preferentially packaged and reverse transcribed (or the corresponding DNA containing nucleocapsids are more stable during work-up for Southern blotting [43]); for the transgene vectors, smaller products would have an even greater advantage over the oversized recombinant pgRNAs, explaining their stronger enrichment. Additional experiments will be required to determine the exact nature of these shorter products. Most importantly, however, these data confirmed that also the long recombinant DNA products contained the transgenes. In line with a negative impact of large transgene size on replication, analogous constructs carrying the 942 bp RLuc ORF and the 1653 bp firefly luciferase ORF did not yield Southern blot detectable HBV DNA signals (data not shown).

Overall consistent data were obtained by qPCR for secreted viral DNAs (bar graphs in Fig. 4A,B). Accordingly, the BsdR vector produced about 30% and the hrGFP vector about 0.5–1% the amounts of the wild-type HBV vector. Copy numbers, given as log10 per 1 ml of culture supernatant, for HepG2 cells were 8.73±0.36 for pCH-9/3093, 8.16±0.24 for the BsdR vector, and 6.72±0.20 for the hrGFP vector. For Huh7 cells the corresponding values were 9.15±0.23, 8.65±0.26, and 6.86±0.15 (n = 3). All differences between the groups were significant, with *P*<0.01 for all pairs except BsdR vector vs. wild-type HBV vector (*P*<0.05). Together these data demonstrated substantial replication capacity of the recombinant BsdR containing HBV vector, and they suggested that a certain size limit of a transgene, somewhere between the 399 bp BsdR gene and the 720 bp hrGFP gene, may not be exceeded without severely compromising replication.

### Physical proof for formation of enveloped BsdR HBV vector particles

**Figure 5:**
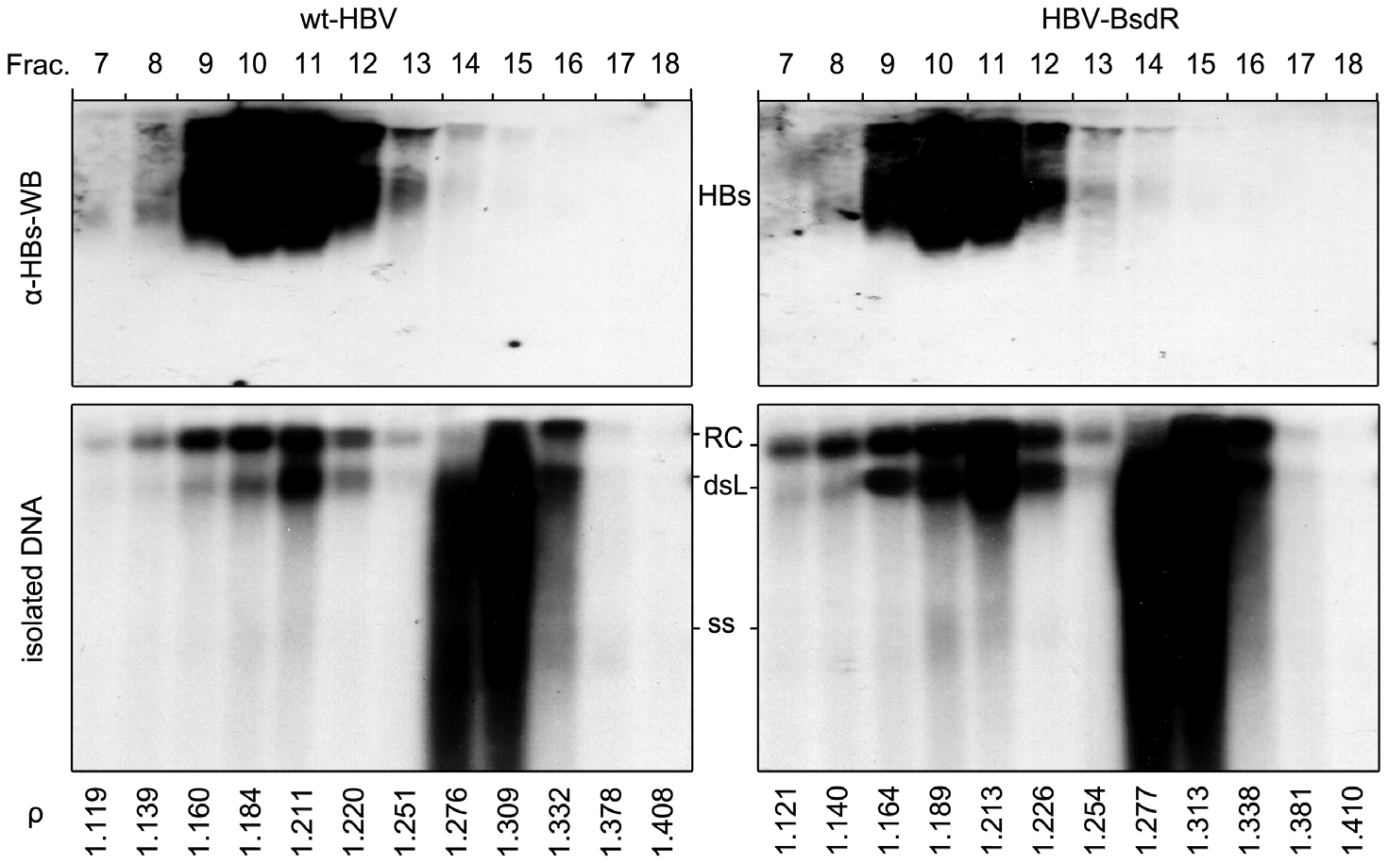
Formation of enveloped virions by the BsdR HBV vector. HepG2 cells were transfected with plasmid pCH-9/3093 or pCH-BsdR. Four days post transfection, viral particles in the culture supernatants were concentrated by PEG precipitation and subjected to CsCl density gradient centrifugation. Twenty-three fractions were collected from the top. Aliquots from fraction 7–18 (their densities ρ are indicated at the bottom) were analyzed by NAGE and immunoblotting for HBsAg (top), or DNA isolated from the same fractions was analyzed by Southern blotting using a ^32^P labeled HBV probe (bottom). The positions of HBsAg (HBs, top panels), and of HBV RC-DNA and dsL DNA (bottom panels) are indicated. The largely mature DNAs in the low density HBsAg-positive fractions reflect the presence of enveloped virions. Viral DNA in the high density HBsAg-negative fractions originates from non-enveloped particles.

Detection of viral DNA in cell culture supernatants does not unequivocally demonstrate the presence of enveloped virions because transfected cells may also release naked nucleocapsids [46], [40], [47]. However, their different buoyant densities allow for an unambiguous distinction [30], [46], [40], [41]. Therefore, extracellular viral particles from cells transfected with the wild-type HBV plasmid and the BsdR vector were separated by CsCl density centrifugation. Equal aliquots from fractions 7–18 (based on previous analogous experiments [46], [40]) were subjected to NAGE analysis followed by immunoblotting for HBsAg, and from the same fractions viral DNA was isolated and analyzed by Southern blotting (Fig. 5). For both wild-type HBV and the BsdR recombinant HBsAg accumulated in fractions 9–12, corresponding to a buoyant density of around 1.16–1.22 g/cm^3^, as expected. The same fractions contained largely mature RC-DNA and dsL-DNA. Consistent with the data shown in Fig. 3F and Fig. 4, the only difference was a higher proportion of dsL- vs. RC-DNA for the recombinant. A second peak of viral DNAs was present in the higher density fractions 14–16 (density range, 1.27–1.33 g/cm^3^) devoid of HBsAg, with a smear of immature DNA products in fraction 14 and increasingly mature products in the higher density fractions. Hence these products arose from non-enveloped particles. Their density distribution is consistent with earlier data for wild-type HBV from stable cell lines [40] and cells transduced with chimeric adenovirus-HBV virus [46]. Most importantly, these data proved formation of full-genome containing recombinant enveloped BsdR vector virions.

### Infectivity of the replication-competent HBV vector particles

As a final test for the functionality of the recombinant virions we assessed their infectivity for HepaRG cells, the only stable cell line known to be infectable by wild-type HBV, however only after differentiation [5]. For the inoculum, viral particles were concentrated by PEG precipitation from the culture supernatants of HepG2 cells transfected with plasmids pCH-9/3093, pCH-BsdR and pCH-hrGFP, and incubated with pre-differentiated HepaRG cells [5], [48] at a nominal multiplicity of infection (MOI) of 100 viral genomes equivalents (vge) per cell; for the poorly replicating hrGFP recombinant an MOI = 10 had to be used; serum of a chronic HBV carrier served as a further positive control.

**Figure 6:**
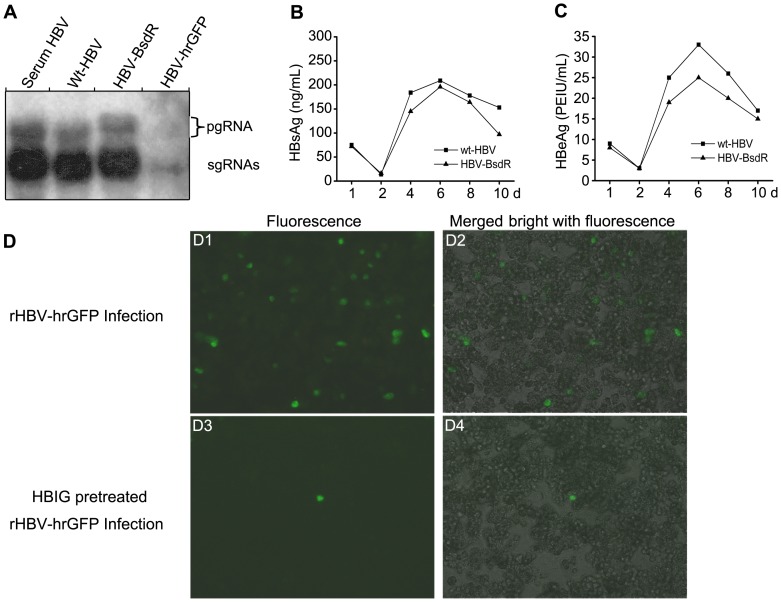
Infection of HepaRG cells by recombinant HBV vector particles. Differentiated HepaRG cells were inoculated with viral particles from culture supernatants of HepG2 cells transfected with the indicated plasmids, or with patient serum-derived HBV, at a nominal moi of 100 vge per cell; the hrGFP HBV vector particles had to be used at 10 vge/cell. (A) *De novo* production of vector-specified RNAs. Eight days post inoculation, total cellular RNA was analyzed by Northern blotting with a HBV-specific probe. The positions of authentic and vector-derived pgRNAs and of the unmodified sgRNAs are indicated. (B,C). Secretory antigen expression. Supernatants from HepaRG cells inoculated with wild-type HBV (▪) or BsdR-HBV vector particles (▴) were analyzed at the indicated time points post inoculation for HBsAg (B) and HBeAg (C). (D) GFP expression in hrGFP-HBV vector inoculated cells indicates successful infection and allows assessment of virus-neutralizing activity. Development of GFP fluorescence in HepaRG cells inoculated with untreated (panel D1) or Hepatitis B immunoglobulin (HBIG) pretreated pCH-hrGFP derived particles (D3) was monitored by fluorescence microscopy. Images were taken at 6 days post inoculation. Merged images of the fluorescent signals with bright field phase contrast are shown in panels D2 and D4, respectively.

Results of a typical infection experiment are shown in Fig. 6. As HB virions lack appreciable amounts of RNA, formation of HBV transcripts indicates successful infection [16]. All inocula except the hrGFP particles led to the easily detectable presence of genomic and subgenomic viral RNAs 8 days post-inoculation (Fig. 6A). The signal intensities from the BsdR vector matched those from cell culture- and serum-derived wild-type HBV; as in transfected cells the recombinant pgRNA displayed reduced mobility. Infection by and active viral replication of the BsdR vector particles were further supported by a wild-type HBV-like net increase of secreted HBsAg and HBeAg (Fig. 6B,C). For wild-type HBV infection of HepaRG cells it is established that transcription requires nuclear cccDNA formation and that HBx is required to ensure transcriptional activity of the cccDNA [15], [16]. Hence formation of wild-type HBV-like amounts of viral RNAs and antigens from the BsdR vector further implies that it also meets these requirements. For the low titered hrGFP vector inoculum, even the subgenomic RNA signals on the Northern blot were very weak; some larger products were also present but, in contrast to transfected cells (Fig. 3B), their sizes could not unambiguously be determined due to their very low abundance. As an independent test for successful infection, we therefore monitored the inoculated cells for development of hrGFP fluorescence. Indeed, fluorescence was observed starting on day 4 post inoculation, and its intensity, though not the fraction of positive cells, gradually increased with time (Fig. 6D1, D2; day 6 p.i.). However, even wild-type HBV shows only very limited or no spreading of infection in HepaRG cells [15]. Importantly, the low level of infection by the hrGFP recombinant still provided a simple means to further test infection specificity by preincubation of the inoculum with wild-type HBV neutralizing hepatitis B immunoglobulin (HBIG). This pretreatment drastically reduced the number of GFP positive cells (Fig. 6D3, D4), indicating that infection by the untreated particles was indeed mediated by the viral envelope proteins, as in wild-type HBV infection. These data also provide a first example for the application of the new vectors to address antiviral activities.

## Discussion

Replication-competent viral vectors carrying additional genetic information have become invaluable tools to monitor and quantitate intracellular replication and, if infectious, the efficiency and route of infection of the parental viruses. Prominent examples include the selection of replication-competent HCV subgenomic replicons via inserted resistance markers [49], and of infectious fluorescent reporter-carrying HCV chimeras [50], [51]. The same has been achieved for other important virus families, including DNA viruses [52], influenza virus [53], [54] and HIV-1 [8], [55], [9], making these vectors also highly useful for drug screening. Viral vectors may also deliver transgene-encoded effector molecules such as shRNAs, cytokines, or toxic principles into cells according to their natural (or appropriately engineered) host and tissue tropism; an example are oncolytic viruses for cancer therapy [56].

Despite its relevance as a human pathogen, attempts to generate replication-competent, let alone infectious, HBV-based vectors had met with very limited success (see Introduction). Our study demonstrates that by careful design of the insertion site and utilization of very small regulatory elements the main obstacle, i.e. the extremely compact organization of the HBV genome, can principally be overcome, allowing production of HBV vectors carrying at least around 400 bp and possibly up to 720 bp of foreign genetic information yet maintaining replication competence and even infectivity. Because numerous reporter and effector genes do not exceed this size limit, these new vectors should become highly useful novel tools for basic and applied HBV research. Below we discuss the pertinent features enabling this advance, the remaining limitations of this first generation of replication-competent HBV vectors and ways for improvement, and provide some examples for potential applications.

### Key features enabling replication-competence and infectivity of the new HBV vectors

Based on current knowledge substantial replication-competence of an HBV vector can only be achieved by preserving all viral gene products and cis-elements required for reverse transcription, i.e. Pol, core protein and a recombinant pgRNA that still can be encapsidated and converted into RC-DNA. For infectivity, on top, the envelope proteins as well as HBx have to be produced at near-normal levels. Hence we fully maintained the viral genetic information and uncoupled Pol translation from core protein translation to create an artificial transgene insertion site with an expected minimal impact on replication competence. Principal size limitations (discussed in more detail below) excluded the use of well-established viral IRES elements, hence we resorted to the 22 nt Rbm3 IRES sequence [36] although its mechanism of action is not well understood.

In the bicistronic model constructs the Rbm3 element gave higher levels of downstream cistron expression than the EMCV IRES, and the third cistron was still reasonably expressed when an additional cassette containing its own upstream Rbm3 IRES was inserted (Fig. 1A,B). Notably, formation of a translational fusion between the second and third cistron giving rise to GFP fluorescence or RLuc activity is highly unlikely because the second cistron is followed by stop codons in all three reading frames before the third cistron starts.

The formation of viral pgRNAs exceeding the size of wild-type HBV pgRNA and the virtual absence of aberrantly sized transgene containing RNAs (Fig. 3B) also argues against splicing [37] as the major underlying principle, although a low level of splicing may occur (see below). Furthermore, Pol has a marked preference for packaging the same RNA it was translated from [57], thus the predominant detection of longer-than-wild-type RC-DNA and dsL-DNA from the BsdR vector (Fig. 3F, Fig. 4A,B, Fig.5) indicates that Pol had indeed been translated from the recombinant pgRNA. Hence the most plausible explanation is that the Rbm3 IRES elements in the tricistronic model constructs, and analogously in the transgene carrying HBV vectors, act by somehow recruiting ribosomes, as proposed [36].

Transfection of the HBV vector plasmids (Fig. 2) into Huh7 and HepG2 confirmed the production of all major viral RNAs and protein products in wild-type HBV-like amounts (Fig. 3), including intact capsids and the full set of envelope proteins (Fig. 3E). Pol expression was proven by endogenous polymerase activity for the recombinant carrying the smaller (399 bp) BsdR though not the larger (720 bp) hrGFP transgene, despite similar levels of recombinant pgRNA and capsids. This would be consistent with defects in Pol expression, and/or packaging or reverse transcription of the recombinant pgRNA which we regard as the most likely explanation (see below). However, Southern blotting (Fig. 4) revealed small amounts of replicative DNA intermediates also for the hrGFP vector, even though size-increased RC-DNA could not unequivocally be detected. Importantly, however, and consistent with the endogenous polymerase assays the BsdR vector produced substantial amounts of size-increased RC-DNA and dsL-DNA; transgene presence was directly demonstrated using transgene-specific probes. These also revealed the presence of minor amounts of faster migrating DNAs, including distinct species (labeled by asterisks in Fig. 4A) which were not detected using by the HBV probe. A likely explanation is that low levels of spliced recombinant pgRNAs, not detectable by Northern blotting (Fig. 3B), are more efficiently packaged and reverse transcribed than their larger-than-wild-type unspliced counterparts, leading to an enrichment of the short DNAs. For wild-type HBV, efficient formation of shortened genomes from spliced pgRNAs is well established; of note, splicing can be ablated by a single point mutation in the common splice acceptor site [43]. Further characterization of the shorter vector DNA products will allow to identify, and knock-out, the corresponding splicing-relevant sites on the recombinant vector genomes. Importantly, however, the bulk of transgene-specific Southern blot signals arose from full-length DNAs, as is most obvious from the short exposures for the BsdR vector (Fig. 4A, lower panel). These data were confirmed by qPCR (Fig. 4) which further suggested that the BsdR vector, and less so the hrGFP vector, were able to direct formation of secreted virions. Density gradient analysis of the extracellular BsdR vector particles proved this assumption (Fig. 5). Infectivity of the recombinant virions was finally demonstrated using differentiated HepaRG cells [5], [48]. The net production of wild-type HBV-like levels of subgenomic and, size-increased, pgRNA from the BsdR recombinant virions (Fig. 6A) strongly suggest efficient infection by the vector particles, as corroborated by the net increase in HBsAg and HBeAg (Fig. 6B). Because cccDNA is considered as the exclusive template for all viral transcripts and its transcriptional activity appears to depend on HBx [15], [16], [58], the data further imply formation of sufficient quantities of recombinant cccDNA and HBx from the recombinant vector genome. Altogether, these results demonstrate that the redesigned HBV vector genome including around 400 bp of additional genetic information is fully functional.

Lastly, the development of GFP fluorescence in cells inoculated (at necessarily low moi) with the hrGFP-recombinant particles demonstrated that even a vector with much reduced replication capacity can still yield a useful reporter readout; in particular, the fluorescence signals were strong enough to clearly visualize the reduction in reporter-positive cells caused by pre-incubation with HBIG, known to neutralize wild-type HBV (Fig. 6C). It is easily imagined that similar assays can be used to screen for inhibitors that affect entry and infection, or intracellular replication. Preliminary data indicate indeed that the presence of the reverse transcriptase inhibitor lamivudine reduces GFP fluorescence intensity per cell but not the fraction of positive (i.e. infected) cells; this is in line with data for wild-type HBV infection of HepaRG cells where lamivudine prevented intracellular replication but not infection and initial cccDNA formation [15]. However, more quantitative analyses will be required to confirm this assumption.

### Current limitations and ways for improvement

Production of replicative DNA intermediates dropped sharply (from about 40% to <1% the levels of wild-type HBV) when the transgene size increased from 399 bp (BsdR) to 720 bp (hrGFP); for even longer transgenes (RLuc: 942 bp; firefly Luc 1653 bp) no replication was detectable. Hence the size of the hrGFP transgene likely marks an upper limit for obtaining HBV vectors displaying practically useful levels of replication. Because reverse transcription of hepadnaviruses occurs inside intact nucleocapsid, size constraints for a recombinant HBV genome operate at several levels. As in other icosahedral capsids, e.g. from adenovirus [59], the available inner capsid volume is principally limited. In vitro assembly of RNA-filled recombinant capsids (in the absence of the genuine Pol-dependent pgRNA packaging process) suggests that up to ∼7 kb of RNA might fit into the capsid [28], [29]. This length of nucleic acid also meets another constraint, namely that its negative charges be counterbalanced by at least 0.6–0.7 molar equivalents of positive charges, provided by the multiple Arg residues in the C terminal nucleic acid binding domain of the core protein [60], [29]. We have not explicitly tested whether pgRNAs carrying long transgenes can be packaged; however, their double-stranded reverse transcription products would have twice as many negative charges and even more demanding volume requirements. Hence even if packaged, substantially extended pgRNAs would likely fail to be reverse transcribed, or possibly cause premature dissociation of the capsid shell. This would also be consistent with the relative enrichment of short transgene-containing DNAs (Fig. 4A, lower panel) over the corresponding RNAs (Fig. 3B). A third constraint are the intricate template switches required to generate RC-DNA which are controlled by numerous cis-elements; in particular, some of them act via long-range interactions [61], [62] which might be disturbed by some foreign sequences via formation of alternative, nonproductive structures. This implies that specific sequence features, in addition to length, might interfere with proper replication. Lastly, length and sequence of a given transgene might also impact Pol translation efficiency from the downstream Rbm3 IRES element, such that Pol becomes limiting for pgRNA packaging and reverse transcription. All these factors may contribute to the low levels of transgene-carrying DNA products from the hrGFP vector. However, our preliminary data for vectors carrying other around 500 bp inserts suggest that such sequence-dependent interference is not widespread. Hence numerous small to medium-sized transgenes should be compatible with replication competence.

A biosafety-relevant aspect of the new vectors is the potential for homologous recombination via the sequence duplications required for uncoupled core and Pol expression and the two identical Rbm3 IRES elements. While we did not see evidence for such events, recombination could recreate wild-type HBV. Hence the described first-generation replication-competent vectors should be handled in accord with the same biosafety regulations that apply to wild-type HBV. However, an easy way to minimize homologous recombination should be to redesign the coding sequences for the carboxy-terminal end of the core protein and the N terminal region of Pol via silent codon exchanges that minimize nt sequence identity. Regarding the duplicated IRES, also other short translation initiation-mediating elements have been described [63], one of which may be used to replace one of the two Rbm3 elements.

### Final conclusions and outlook

This study demonstrates how by careful redesign of its intricate genome organization HBV can be harnessed into a replication-competent infectious vector bearing substantial additional genetic information. Numerous available reporter and effector genes meet the apparent size limit of 500–700 bp. For instance, the *Gaussia princep*s luciferase coding sequence encompasses only 555 bp [64] and thus is only slightly larger than the BsdR ORF shown here to provide high replication competence. Other antibiotic resistance genes, e.g. against bleomycin are even smaller (less than 400 bp; [65]). MiniSOG is an engineered fluorescent protein of only 106 aa which can be visualized by fluorescence and electron microscopy, and be used as an optogenetic tool via singlet oxygen generation [66], [67]. Truncated SV40T antigens comprising around 150 aa are able to immortalize cells [68]. Given their strict hepatocyte tropism, HBV vectors equipped with appropriate transgenes can thus be expected to find numerous applications, from further unravelling the molecular mechanism of HBV infections, including the involved host factors, to the identification of infectable cells and new antivirals as well as the specific manipulation of hepatocytes.

## Materials and Methods

The sequences of the individual primers used to generate the plasmid constructs in Fig. 1 are reported in Table S1, and the exact IRES-flanking sequences in vectors pCH-BsdR and pCH-hrGFP in Table S2.

### Plasmid constructs

The parental HBV plasmids used were pCH-9/3093 [40], [41], with a 2 nt deletion compared to its predecessor pCH-9/3091 [30] in the non-transcribed HBV sequence immediately following the CMV-IE enhancer promoter, and pHBV1.3 [46], carrying a 1.3× genome length HBV genome in which pgRNA transcription is controlled by the authentic HBV core promoter/enhancer II; both plasmids harbor a genotype D, subtype ayw HBV isolate of proven infectivity (Genbank accession: V01460.1 [69]).

Two series of model plasmids for testing the activity of the Rbm3 IRES were constructed. The first was driven by the CMV-IE enhancer/promoter and encoded enhanced green fluorescent protein (EGFP) as marker for visual inspection (Fig. 1A); the second was driven by the endogenous HBV core promoter/enhancer II and encoded *Renilla* luciferase (RLuc) for expression quantification (Fig. 1B). For tricistronic constructs, a Blasticidin resistance (BsdR) gene bringing its own Rbm3 IRES was inserted between the upstream and downstream cistron of the bicistronic constructs. All were assembled from two or three, respectively, PCR products obtained using Phusion High-Fidelity DNA Polymerase (Fermentas). For the backbone parts, plasmids pCH-9/3093 and pHBV1.3 served as templates. The EGFP and RLuc ORFs including the poly(A) signals were amplified from pEGFP-N1 (Clontech) and pGL4.79 (Promega) respectively. The EMCV IRES element was amplified from a hepatitis C virus replicon vector (kind gift of B. Yen, USA), the 22-nt Rbm3 IRES element was chemically synthesized. The specific sequence was TTT ATA ATT TCT TCT TCC AGA A GA ATT TGT TGG TAA AGC CAC C**ATG** ; the underlined part represents the IRES element, the following sequence a linker for connection to the downstream ATG (bold face) [36]. The primers for construction of the 8 plasmids are provided in Table S1. For construction of pCH-BsdR, the three PCR products PCR1 (template pCH-9/3093; primers HBV1.05-S: CAA TTG TCG ACA CCA TGC AAC TTT TTC ACC TCT GCC TA and 22ntIRES-Core-AS: CTT GGC CAT GGT GGC TTT ACC AAC AAA TTC TTC TGG AAG AAG AAA TTA TAA ATT AAC ATT GAG ATT CCC GAG A), PCR2 (a BsdR gene containing plasmid as template; primers BsdR-S: CGC CAC CAT GGC CAA GCC TTT GTC TC and BsdR-AS: GCG CAC TGC AGT TAG CCC TCC CAC ACA TAA), and PCR3 (template pCH-9/3093; primers 22ntIRES-P-S: GCG CGC TGC AGA AAT TTA TAA TTT CTT CTT CCA GAA GAA TTT GTT GGT AAA GCC ACC ATG GCC CTA TCC TAT CAA CAC and HBV1549-AS: GAA GTC CAC CAC GAG TCT AGA CTC) were digested with restriction enzymes using specific sites provided by the primers, and the purified fragments were ligated. The product served as template for amplification with the terminal primers HBV1.05-S and HBV1549-AS, and the resulting PCR product was digested with Sal I and Eco RI; this was then used to replace the authentic HBV Sal I - Eco RI fragment in pCH-9/3093. All sequences were confirmed by DNA sequencing. For construction of pCH-hrGFP, the BsdR gene was replaced by the humanized *Renilla* GFP (hrGFP) gene (Stratagene) via the 5′ terminal Nco I and blunt-ended 3′ terminal Pst I sites. The resulting junction sequences around the upstream and downstream Rbm3 IRES elements in these vectors are shown in Table S2.

### Cell culture and transfection

Huh7 and HepG2 cells were cultured as previously described [40]. Transfections were performed using Fugene HD reagent as recommended by the manufacturer (Roche). Cytoplasmic lysates and supernatants were harvested between day 3 and 5 post-transfection, as indicated in the text.

### Isolation of viral nucleic acids, Southern and Northern blotting, quantitative PCR (qPCR)

Cytoplasmic lysates, as a source of intracellular viral nucleocapsids, were prepared using lysis buffer (10 mM Tris-HCl PH 7.5, 50 mM NaCl, 1 mM EDTA, 0.25% Nonidet P-40, 8% saccharobiose) as previously described [40]. Extracellular viral particles were precipitated by polyethylene glycol (PEG) 8000. Cytoplasmic and extracellular HBV DNA were isolated after DNase I, RNase A and micrococcal nuclease treatment to remove RNA and plasmid DNA, and SDS and proteinase K followed by phenol/chloroform extraction and isopropanol precipitation. Viral DNAs were detected by Southern blotting using a random-primed [α-^32^P]dCTP labeled HBV probe [70] or analogously prepared BsdR- and hrGFP-specific probes. ^32^P signals were quantified using a PhosphoImager and Kodak Multi Gage software. Cytoplasmic RNAs was isolated by the SV Total RNA Isolation System Kit (Promega) and analysed by Northern blotting using the same ^32^P labeled HBV- or transgene-specific probes. For qPCR, aliquots of prepurified extracellular virals were treated by Dpn I to digest any residual transfected plasmid DNA. Viral titers were measured using a commercial HBV DNA kit (Kehua, Shanghai, China) on a SLAN™ Real-Time PCR system (Hongshi, Shanghai, China).

### Immunoblotting

Immunoblotting after SDS-PAGE was performed as described [40], [70], using human monoclonal antibody 4/7B [71] to detect HBV envelope proteins, and antibodies mc158 and mc312 [72] conjugated with peroxidase (PO) to detect core protein and capsids. Intact capsids were separated by native agarose gel electrophoresis (NAGE) followed by immunoblotting [46], [40]. Nucleocapsid-packaged DNA was released via brief treatment with 0.1 M NaOH and detected by hybridization with the ^32^P labeled HBV DNA probe.

### Buoyant density separation of viral particles

Separation of PEG 8000 enriched viral particles from culture supernatants was done as described [46], [40]. Briefly, the 1.5 ml viral particle samples were loaded on 10 ml cesium chloride (CsCl) step gradients and centrifuged at 32,000 rpm/min for 35 h at 4°C in a Kontron TST 41.14 rotor. Twenty-three 0.5 ml fractions were then collected from the top. Densities were determined via refractive index. Fractions 7–28 were analyzed for HBsAg by NAGE analysis and immunoblotting using antibody 9H9 [71], and for viral DNA by Southern blotting.

### Endogenous polymerase assays

Endogenous polymerase assays were carried out as previously described, with minor modifications [73]. Cytoplasmic lysate was prepared three days post transfection. DNase I and RNase A were added to remove RNA and transfected plasmid DNA. HBV virions were precipitated using 6% PEG 8000, and then were resuspended in endogenous polymerase reaction buffer (50 mM Tris HCl [pH 8.0], 0.1% Nonidet P-40,40 mM MgCl_2_, 50 mM NaCl, 0.3% β-mercaptoethanol, 2 mM of each of dGTP, dATP, and dTTP and 10 µCi of [α-^32^P]dCTP) and incubated for 2 h at 37°C. Reactions were stopped by adding EDTA, proteinase K and SDS and incubated at 45°C for another 2 h. Viral DNA was extracted with phenol-chloroform and precipitated using sodium acetate and ethanol. ^32^P-labeled viral DNAs were separated by electrophoresis in 1% agarose gels and detected by autoradiography.

### Infection of HepaRG cells

HepaRG cells were obtained from Biopredic International (Rennes, France). The culture and differentiation conditions were as described [5], except that prior to infection, the differentiated cells were preincubated for 30 minutes with 800 µM of ethylene glycol tetraacetic acid (EGTA) as recently described [48]. For infection, viral particles were collected from the culture supernatants of pCH-9/3093 transfected HepG2 cells, or human HBV-positive serum by PEG 8000 precipitation. For blocking infection, the viral particles were preincubated with Hepatitis B immunoglobulin (HBIG, 2 IU per 10^6^ cells, Tonrol Biological Pharmaceutical Co., Ltd. China), for 1 h at room temperature as described [74], followed by incubation with the cells for 24 hours; thereafter, the cells were washed and culture medium was exchanged every second day.

### Luciferase assay, HBsAg and HBeAg ELISA

For determination of relative RLuc activity, cells were cotransfected with the respective RLuc vectors and the firefly luciferase vector pGL3-Control (Promega). Thirty-six hours post-transfection, luciferase activities were measured by the dual luciferase assay as recommended by the manufacturer (Promega). HBsAg and HBeAg in cell culture supernatants were assayed using commercial ELISA kits (Chemclin Biotech Co., Ltd. Beijing, China).

### Statistical analysis

All data were expressed as mean ± standard deviation (SD). SPSS18.0 software was used for statistical analysis. Comparisons between two and multiple groups were made using Student's t test and One-way ANOVA, respectively. Values of *P*<0.01 or *P*<0.05 were regarded as statistically significant.

## Supporting Information

Table S1 **Primers used for constructing the plasmids shown in** **Fig. 1** **.**
(DOC)

Table S2 **Detailed junction sequences around the upstream and downstream Rbm3 IRES elements in vectors pCH-BsdR and pCH-hrGFP.**
(DOC)
