# Supplementary material for: Replication-Competent Infectious Hepatitis B Virus Vectors Carrying Substantially Sized Transgenes by Redesigned Viral Polymerase Translation
Source: PLoS One. 2013 Apr 2;8(4):e60306. doi: 10.1371/journal.pone.0060306 (PMC3615001; doi:10.1371/journal.pone.0060306)
Supplement: Table S1 — Primers used for constructing the plasmids shown in Fig. 1 . (DOC) [file pone.0060306.s001.doc]

**SUPPORTING INFORMATION**

**Table S1**

**Primers used for constructing the plasmids shown in Fig. 1^§^.**

HBV1.3-S: GCGCG**AAGCTT**GTAATCATGGTCATAGCTGTTTC

CMV-S: GCGCG**AAGCTT**CCTATAGTGAGTCGTATTAATA

CoreEND-AS: CGCGC**CTTAAG**CTAACATTGAGATTCCCGAGATTG

CorePART-AS: TTGGTGGTCTATAAGCTGG (5' phosphorylated)

EGFP-S: CACCGGTCGCCA**CCATGG**

EGFP-PA-AS: GCGCG**AAGCTT**AAGATACATTGATGAGTT

hLuc-S: GTTGGTAAAGCCA**CCATGG**

hLuc-PA-AS: CGCGC**AAGCTT**GATTTTACCACATTTGTAGAGG

EGFP-ATG-S: ATGGTGAGCAAGGGCGAGGA (5' phosphorylated)

hLucATG-S: ATGGCTTCCAAGGTGTACGAC (5' phosphorylated)

EMCV-S: CGCGT**CTTAAG**TTTAAACAGACCACAACGG

EMCV-AS: GCGCA**CCATGG**TATTATCGTGTTTTTC

22ntIRES-S: TTAAGTTTATAATTTCTTCTTCCAGAAGAATTTGTTGGTAAAGCCAC

22ntIRES-AS: CATGGTGGCTTTACCAACAAATTCTTCTGGAAGAAGAAATTATAA AC

Bsd-S: CGCCA**CCATGG**CCAAGCCTTTGTC

Bsd-AS: GCGCA**CTTAAG**TTAGCCCTCCCACACATAA

^§^ All primers are shown in 5´>3´ direction. Primer-encoded restriction sites used for cloning are in bold-face and underlined. Templates for individual PCR reactions using these primers are outlined in Materials and Methods.
