# Supplementary material for: Replication-Competent Infectious Hepatitis B Virus Vectors Carrying Substantially Sized Transgenes by Redesigned Viral Polymerase Translation
Source: PLoS One. 2013 Apr 2;8(4):e60306. doi: 10.1371/journal.pone.0060306 (PMC3615001; doi:10.1371/journal.pone.0060306)
Supplement: Table S2 — Detailed junction sequences around the upstream and downstream Rbm3 IRES elements in vectors pCH-BsdR and pCH-hrGFP. (DOC) [file pone.0060306.s002.doc]

**SUPPORTING INFORMATION**

**Table S2**

**Detailed junction sequences around the upstream and downstream Rbm3 IRES elements in vectors pCH-BsdR and pCH-hrGFP^§^.**

**pCH-BsdR**

Core end - IRES - BsdR start

caa tgt **taa-**TTTATAATTTCTTCTTCCAGAAGAATTTGTTGGTAAAGCCACC-**atg** gcc aag

BsdR end - IRES - Pol start

agg ggc taa-CTGCAGAAATTTATAATTTCTTCTTCCAGAAGAATTTGTTGGTAAAGCCACC-**atg g**cc cta

**pCH-hrGFP**

Core end - IRES - hrGFP start

caa tgt taa-TTTATAATTTCTTCTTCCAGAAGAATTTGTTGGTAAAGCCACC-**atg** gtg agc

hrGFP end - IRES - Pol start

gtc tag-AGAAATTTATAATTTCTTCTTCCAGAAGAATTTGTTGGTAAAGCCACC-**atg g**cc cta

^§^The sequences in lower case show the last two sense codons of the upstream ORFs (stop codon in red) and the first three codons of the downstream ORF (start atg in green). The red g following the Pol start codon indicates a C>G exchange for an optimized Kozak consensus (G at position +4). The Rbm3 IRES element sequence is underlined. Additional TAA stop codons for preventing readthrough from the respective upstream ORFs are highlighted in red.
